# Mendelian randomization study of circulating lipids and biliary tract cancer among East Asians

**DOI:** 10.1186/s12885-022-09382-x

**Published:** 2022-03-15

**Authors:** Jun Wang, Jinke Zhuge, Dongxu Feng, Bo Zhang, Jianying Xu, Dongkang Zhao, Zhewei Fei, Xia Huang, Wenjie Shi

**Affiliations:** 1 Department of General Surgery, Xinhua Hospital Chongming Branch, 25 Nanmen Road, ShanghaiChongming, 202150 China; 2 Department of Respiratory Medicine, Hainan Cancer Hospital, Haikou, 570311 Hainan China; 3 Department of Medical Oncology, State Key Laboratory of Oncology in South China, Collaborative Innovation Center for Cancer Medicine, Sun Yat-Sen University Cancer Center, Guangzhou, 510060 China; 4 Department of Hepatobiliary Surgery, The Second Affiliated Hospital of Guilin Medical University, Guilin, 541100 Guangxi China; 5 University Hospital for Gynecology, Pius-Hospital, University Medicine Oldenburg, 12 Georg Street, 26121 Oldenburg, Germany

**Keywords:** Biliary tract cancer, Mendelian randomization, Lipid, HDL, LDL, Triglyceride, Cholesterol

## Abstract

**Background:**

Associations of High-density lipoprotein (HDL) cholesterol, low-density lipoprotein (LDL) cholesterol, total cholesterol (CHL), and triglyceride (TRG) concentrations with risk of biliary tract cancer (BtC) were conflicting in observational studies. We aim to investigate the causal link between circulating lipids and BtC using genetic information.

**Methods:**

Single nucleotide polymorphisms of the four circulating lipids (*n* = 34,421) and BtC (418 cases and 159,201 controls) were retrieved from two independent GWAS studies performed in East Asian populations. Two-sample univariate and multivariate Mendelian Randomization (MR) analyses were conducted to determine the causal link between circulating lipids and BtC.

**Results:**

No significant horizontal pleiotropy was detected for all circulating lipids according to the MR-PRESSO global test (*P* = 0.458, 0.368, 0.522, and 0.587 for HDL, LDL, CHL, and TRG, respectively). No significant evidence of heterogeneity and directional pleiotropy was detected by the Cochran’s Q test and MR-Egger regression. Univariate MR estimates from inverse variance weighting method suggested that one standard deviation (1-SD) increase of inverse-normal transformed HDL (OR = 1.38, 95% CI 0.98–1.94), LDL (OR = 1.46, 95% CI 0.96–2.23), and CHL (OR = 1.34, 95% CI 0.83–2.16) were not significantly associated with BtC risk. Whereas 1-SD increase of inverse-normal transformed TRG showed a significantly negative association with BtC risk (OR = 0.48, 95% CI 0.31–0.74). In multivariate MR analyses including all the four lipid traits, we found that 1-SD increase of LDL and TRG was significantly associated with elevated (OR = 1.32, 95% CI 1.04–2.01) and decreased (OR = 0.54, 95% CI 0.42–0.68) risk of BtC, respectively.

**Conclusion:**

Circulating lipids, particularly LDL and TRG, may have roles in the development of BtC. However, the results of this study should be replicated in MR with larger GWAS sample sizes for BtC.

**Supplementary Information:**

The online version contains supplementary material available at 10.1186/s12885-022-09382-x.

## Introduction

Biliary tract cancer (BtC) constitutes approximately 3% of gastrointestinal malignancies with poor prognosis and involves a spectrum of invasive adenocarcinomas, including cholangiocarcinoma (cancers arising in the intrahepatic, perihilar, or distal biliary tree), and gallbladder carcinoma [1, 2]. The incidence of BtC varies across the world: the highest incidence rate was observed in East Asia and Latin America [2, 3]. In developed countries, BtC was rarely diagnosed in clinical practice. The varied BtC incidences in different regions were due to different underlying risk factors. Previous studies have demonstrated that a set of hepatic conditions including hepatic inflammation, fibrosis, and cirrhosis are risk factors for intrahepatic cholangiocarcinoma [4]. On the other hand, chronic irritation or inflammation of the gallbladder and cholelithiasis are deemed to be associated with a higher risk of gallbladder carcinoma [5–7]. Additionally, hyperlipidemia was also reported to associate with BtC development even after adjustment for body-mass index (BMI), diabetes, hypertension, and alcohol drinking [8, 9].

Hyperlipidemia is characterized by high serum levels of total cholesterol (CHL), triglycerides (TRG), low-density lipoprotein cholesterol (LDL), and low level of high-density lipoprotein cholesterol (HDL). Previous observational studies suggested a role for the circulating lipids in biliary carcinogenesis. For example, Andreotti et al. reported that participants in the lowest quintile of serum HDL level had a 16.8-fold risk of BtC [9]. Another case–control study from China also suggested that serum levels of lipids were significantly associated with BtC risk [10]. However, the findings from observational studies might be subject to the inherent defects of this type of study design, namely residual confounding and reverse causality. So far, there has been no randomized clinical trial to assess the effect of statin use on BtC development. In this case, Mendelian randomization (MR) analysis could serve as a good surrogate. MR leveraging genetic data is less susceptible to such biases due to the fact that alleles are randomly assigned during meiosis and germline genetic variants are unaffected by disease process [11]. So far, MR analysis has been widely used to infer the causality between exposures and outcomes [12–14]. The findings of MR studies were of importance not only for the discovery of disease biomarkers, but also for the therapeutic and prophylactic strategies of diseases [15]. Nevertheless, the association of circulating lipids with BtC risk has not been determined by MR analysis. Herein, we conducted a two-sample MR analysis to address this need.

## Methods

### GWAS summary statistics of circulating lipids

We collected the GWAS summary data of circulating lipids from the Asian Genetic Epidemiology Network (AGEN; https://blog.nus.edu.sg/agen/). AGEN is a consortium of genetic epidemiology studies of type 2 diabetes and cardiovascular disease related phenotypes including HDL, LDL, CHL, and TRG conducted among East Asian populations [16]. Plasma lipid levels were measured by standard biochemical methods [16]. In the GWAS of circulating lipids, 34,421 participants from China, Japan, Korea, Philippines, and Singapore were included. The participants were genotyped using commercially available Affymetrix or Illumina genome-wide genotyping arrays, and the genotype data were then imputed to HapMap Project Phase II reference panel. Quality control criteria implemented in each population, including variant call rate and Hardy–Weinberg equilibrium (HWE). The GWAS details have been shown elsewhere [16]. Briefly, in GWAS of circulating lipids, age, age^2^, sex, and other study-specific covariates (e.g., principal components, sample recruitment sites) were adjusted in a linear regression model. The levels of circulating lipids (mg/dL) have been normal-inverse transformed in the GWAS. A meta-analysis for associations between the four lipid traits and ~ 2.4 million variants were then performed by two independent analysts, each using Stouffer sample-size weighted fixed effects meta-analysis implemented in METAL.

### GWAS summary statistics of biliary tract cancer

To ensure the concordance of ancestry of study participants, in this study, we retrieved the GWAS summary data of BtC from Biobank Japan (BBJ) [17]. BBJ is a prospective genome biobank that collaboratively collected DNA and serum samples from 12 medical institutions in Japan, managed by the Institute of Medical Science, the University of Tokyo. BBJ has recruited approximately 260,000 participants, mainly of Japanese ancestry. All study participants had been diagnosed with one or more of 47 target diseases, among which the BtC was identified using ICD-10 codes of C22.1 and C23 and ICD-9 codes of 155 and 159.3. The BBJ participants were genotyped with the Illumina HumanOmniExpressExome BeadChip or a combination of the Illumina HumanOmniExpress and HumanExome BeadChips [18]. The genotype data were then imputed with 1000 Genome Project Phase 3 version 5 genotype and Japanese whole-genome sequencing data (*n* = 1037). Variants with an imputation quality < 0.7 were excluded, resulting in a total of 13,530,797 variants analyzed in the GWAS. For BtC, 418 cases and 159,201 controls that were East Asian ancestry were included (https://pheweb.jp/pheno/BtC). A generalized linear model that performed in SAIGE (version 0.37) was applied to conduct BtC GWAS, where age, age^2^, sex, age × sex, age^2^ × sex, and the top 20 principal components were adjusted.

### Genetic instrumental variables

We conducted a series of quality control steps to select eligible instrumental SNPs of circulating lipids. First, we extracted SNPs showing association with lipid levels at the traditional GWAS threshold (*P* < 5 × 10^–8^). Second, we performed a clumping process (*R*^2^ < 0.01; window size = 10,000 kb) based on the linkage disequilibrium (LD) estimates from the East Asian samples in 1000 genomes project. Among those pairs of SNPs that had LD estimate above the specified threshold (0.01), we only retained the SNP that had the lower *P* value. Third, SNPs with a minor allele frequency < 1% were removed. Next, we extracted the statistics (i.e., beta coefficient and standard error) regarding the above selected SNPs from the BtC GWAS summary. If a particular requested SNP was absent in the BtC GWAS, we retrieved the data of a SNP proxy that had LD estimate *R*^2^ > 0.8 with the requested SNP. The effects of ambiguous SNPs with inconsistent alleles and palindromic SNPs with ambiguous strand were either corrected or directly excluded in the subsequent two-sample MR analysis. The methodological details of MR analysis were presented elsewhere [19, 20].

### Mendelian randomization analysis

**Fig. 1:**
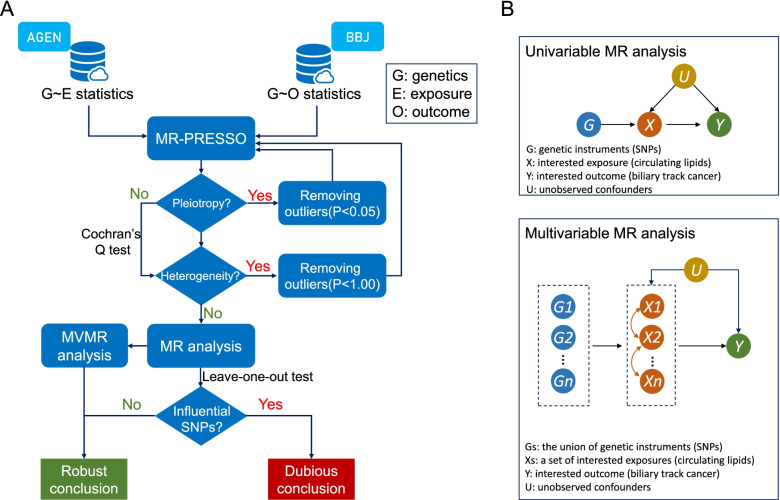
Flow chart (**A**) and schematic representation (**B**) of Mendelian randomization analysis in this study

The flowchart and schematic representation of MR analysis is shown in Fig. 1. First, we tested the horizontal pleiotropy using MR-PRESSO global test and removed the outliers (i.e., SNPs with *P* < 0.05) if the horizontal pleiotropy was presented. Second, we tested the between-SNP heterogeneity using inverse variance weighting (IVW) method based on the SNPs that retained after pleiotropy correction. The Cochran’s Q statistic was used to check for the presence of heterogeneity. In this step, we removed the SNPs with *P* < 1.00 in MR-PRESSO analysis if the heterogeneity was significant (*P* value of Cochran’s Q statistic < 0.05). Third, we conducted MR analysis using IVW method. We obtained the IVW estimate by meta-analyzing the SNP specific Wald estimates using multiplicative random effects. Given the small case number in the BtC GWAS, we calculated the statistical power for MR analysis using mRnd website (https://shiny.cnsgenomics.com/mRnd/) [21]. We also conducted a set of sensitivity analyses using MR-Egger regression, weighted median, and weighted mode methods. The MR-Egger regression is based on the InSIDE (INstrument Strength Independent of Direct Effect) assumption and consists of three parts: (i) a test for directional pleiotropy, (ii) a test for a causal effect, and (iii) an estimate of the causal effect [22]. The weighted median and weighted mode methods are more robust than IVW and MR-Egger methods if more than 50% of SNPs are invalid instruments [23, 24]. Finally, “leave-one-out” analysis was conducted to detect the influential SNPs. To interrogate the presence of reverse causation, we conducted MR analyses in which the BtC was set as exposure and lipids were set as outcomes. In this analysis, we used a *P* value threshold < 5 × 10^–5^ to select the genetic instruments due to there was no SNP reached the traditional GWAS threshold. A total of 42 variants were obtained after data clumping.

Considering the correlations among circulating lipids, we also performed a multivariable MR (MVMR) analysis including all of the four lipid traits to obtain the causal estimates (Fig. 1B). MVMR is an extension of MR that allows for the causal effects of multiple exposures on an outcome to be estimated [25]. MVMR estimates the “direct” causal effects of each exposure included in the estimation on the outcome, conditional on the other exposures included in the model [26]. MVMR is particularly useful when examining the causal effects of several exposures that are correlated with each other. We also incorporated BMI into the MVMR to examine the potential mediation of obesity on association between lipids and BtC risk. The summary genetic data of BMI from East Asians were retrieved from IEU OpenGAWS project (https://gwas.mrcieu.ac.uk/datasets/bbj-a-1/). All statistical analyses were implemented using *TwoSampleMR* and *MRPRESSO* packages in R program (v 3.6.3). *P* value < 0.05 was considered statistically significant.

## Results

After the quality control processes, we included 26, 19, 23, 10 variants in MR analysis for HDL, LDL, CHL, and TRG, respectively (Supplementary Tables S1-4). The mean *F* statistics for every instrument-exposure association were greater than 10 in our study (*F* = 21.2, 13.4, 18.4, and 10.5 for HDL, LDL, CHL, and TRG, respectively), demonstrating the small possibility of weak instrumental variable bias. No significant horizontal pleiotropy was detected for all circulating lipids according to the MR-PRESSO global test (*P* = 0.458, 0.368, 0.522, and 0.587 for HDL, LDL, CHL, and TRG, respectively). The results of assessment of heterogeneity and directional pleiotropy are shown in Table 1. No significant evidence of heterogeneity and pleiotropy was detected by the Cochran’s Q test and MR-Egger regression, suggesting the variants that included in MR analysis are valid instruments.

**Table 1:** Association of circulating lipids with biliary tract cancer risk according to different methods

	**HDL**	**LDL**	**CHL**	**TRG**
**Inverse variance weighted**				
OR (95%CI)	1.38 (0.98, 1.94)	1.46 (0.96, 2.23)	1.34 (0.83, 2.16)	0.48 (0.31, 0.74)
Q statistics (*P* value)	24.6 (0.431)	20.6 (0.298)	25.5 (0.274)	5.9 (0.662)
**MR-egger**				
OR (95%CI)	2.10 (0.99, 4.46)	1.36 (0.70, 2.63)	1.50 (0.50, 4.50)	0.46 (0.19, 1.12)
Q statistics (*P* value)	23.3 (0.444)	20.5 (0.247)	25.5 (0.228)	5.9 (0.556)
Intercept (*P* value)	-0.048 (0.274)	0.009 (0.804)	-0.009 (0.842)	0.004 (0.946)
**Weighted median**				
OR (95%CI)	1.55 (0.94, 2.58)	1.74 (1.02, 2.97)	2.16 (1.15, 4.07)	0.46 (0.27, 0.76)
**Weighted mode**				
OR (95%CI)	1.73 (0.99, 3.02)	1.75 (0.97, 3.14)	2.51 (0.98, 6.40)	0.48 (0.27, 0.83)

*HDL* high density lipoprotein, *LDL* low density lipoprotein, *CHL* cholesterol, *TRG* triglyceride

**Fig. 2:**
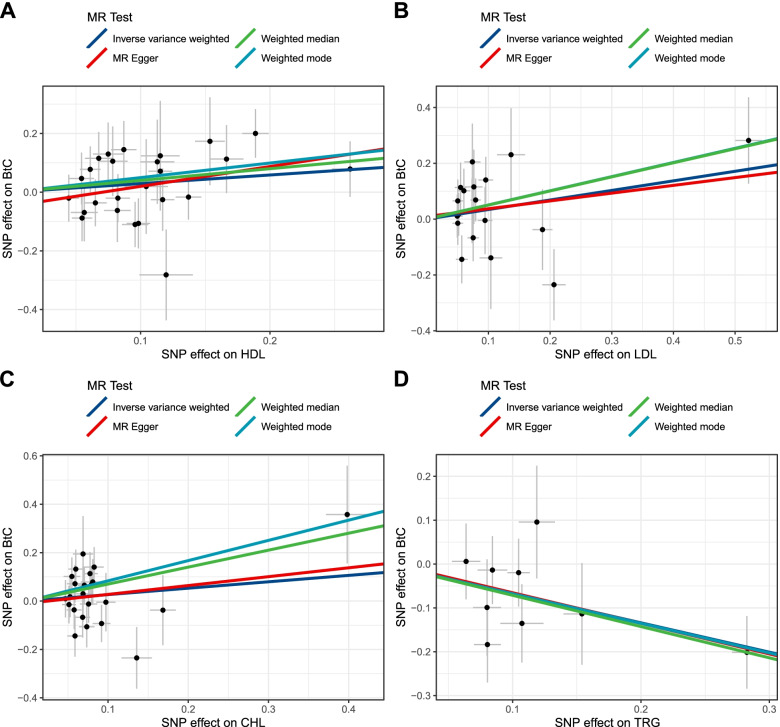
Scatter plots for Mendelian randomization analyses of the causal effect of circulating lipids on biliary tract cancer in initial practice. **A**, HDL; **B**, LDL; **C**, cholesterol; **D**, triglyceride. The slope of each line corresponding to the estimated MR effect per method

**Fig. 3:**
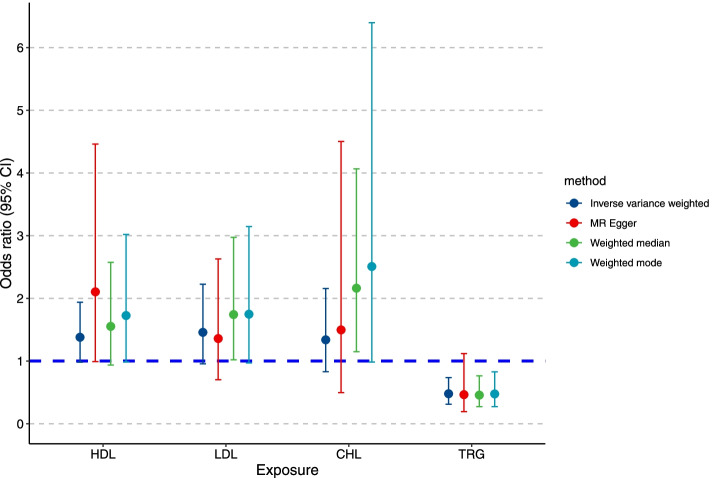
The causal effects of circulating lipids on biliary tract cancer from Mendelian randomization analyses based on four methods. Error bars denote 95% confidence interval of the odds ratio estimates

The estimated effect sizes of the SNPs on both the exposures (HDL, LDL, CHL, and TRG) and outcome (BtC) are displayed in scatter plots (Fig. 2). The fitted lines denoting association between SNP effects on exposure and on outcome, based on different methods, were in the same direction, albeit the nuances of slopes. This concordance connotes the robustness of our MR estimates. MR estimates from IVW method suggested that one standard deviation (1-SD) increase of inverse-normal transformed HDL (OR = 1.38, 95% CI 0.98–1.94), LDL (OR = 1.46, 95% CI 0.96–2.23), and CHL (OR = 1.34, 95% CI 0.83–2.16) were not significantly associated with BtC risk (Table 1; Fig. 3). Whereas 1-SD increase of inverse-normal transformed TRG showed a significantly negative association with BtC risk (OR = 0.48, 95% CI 0.31–0.74). We have calculated 80% power in our MR studies to show an OR of 1.56 for HDL, 1.75 for LDL, 1.59 for CHL, and 0.74 for TRG respectively. As such, we are underpowered to study effects smaller than these ORs. The IVW-based MR estimates were further validated in other three methods. An exception was found for weighted median methods, in which 1-SD increase of inverse-normal transformed LDL and CHL levels were significantly associated with an increased risk of BtC. We observed a non-significant association between 1-SD increase of inverse-normal transformed TRG level and BtC risk according to MR-Egger method, although this association was significant according to other three methods (Table 1; Fig. 3). The forest plots of “leave-one-out” analyses were shown in Supplementary Figs. 1–4. No potentially influential SNP was found for HDL, CHL, and TRG. In contrast, we found that the association between 1-SD increase of inverse-normal transformed LDL level and BtC risk was statistically significant if removing a variant (rs10119). No significant association was detected in MR analysis when examining the BtC effect on levels of circulating lipids (*F* = 4.1, 5.2, 3.8, and 3.5 for HDL, LDL, CHL, and TRG, respectively; Supplementary Table S5). However, the results might be subject to weak instrument bias in this analysis due to the low F-statistics.

**Fig. 4:**
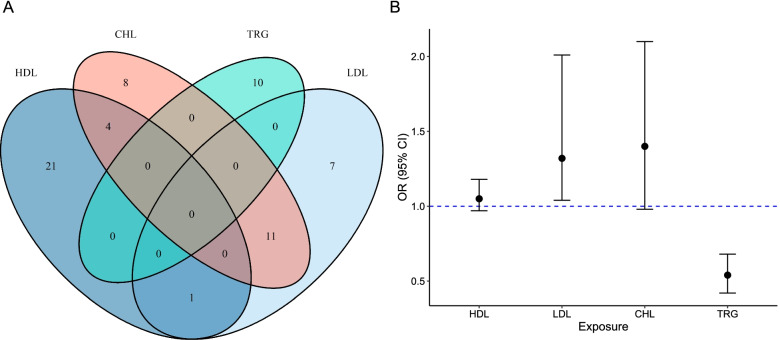
The causal effects of circulating lipids on biliary tract cancer from multivariate Mendelian randomization analyses. **A**, overlap of genetic instruments among the four lipids; **B**, causal estimates from multivariate Mendelian randomization analysis. Error bars denote 95% confidence interval of the odds ratio (OR) estimates

The overlap among genetic instruments of circulating lipids was shown in Fig. 4A. We observed that a total of 11 variants were shared between LDL and CHL, whereas for other pairs of lipids, the shared variants were less than 5. We conducted a MVMR analysis to further validate the association between genetically predicted levels of circulating lipids and BtC risk. MVMR analysis estimated that 1-SD increase of inverse-normal transformed LDL was significantly associated with elevated risk of BtC (OR = 1.32, 95% CI 1.04–2.01). On the contrary, 1-SD increase of inverse-normal transformed TRG was significantly associated with decreased risk of BtC (OR = 0.54, 95% CI 0.42–0.68) (Fig. 4B). We also performed pairwise MVMR analysis between TRG and other three lipids. In all of the three models, we found that TRG were consistently associated with a decreased BtC risk (Supplementary Figure S5). Likewise, we observed an inverse relationship between TRG level and BtC risk in MVMR analysis in which we further incorporated BMI (Supplementary Figure S6).

## Discussion

In this study, using several MR methods, we tested for a causal relationship between circulating lipid traits and BtC risk. Our results suggested that genetically elevated TRG concentration was associated with a decreased risk of BtC. Multivariable MR analysis revealed that genetically elevated LDL level was associated with an increased risk of BtC, although this result did not detect in conventional MR analysis. Our findings were deemed to be robust due to no pleiotropy and heterogeneity was detected and were highly consistent with that of sensitivity analyses.

Biliary tract system plays important roles in many metabolic processes that are critical for the maintenance of body homeostasis [27, 28]. For example, lipid metabolism was reported to closely associate with biliary tract (including gallbladder) [29]. Therefore, it is reasonable to assume that damage in this organ may have a reflection in blood lipids. In other word, alterations of circulating lipid levels may suggest an injury in biliary tract. Indeed, a set of epidemiological studies have reported association of circulating lipid levels with biliary diseases [9, 30, 31]. However, the reported associations were not consistent between studies. For instance, results from previous studies of total CHL and LDL with gallstones are conflicting, with some studies reporting inverse, positive, and null associations [31–35]. Andreotti et al. reported that participants with the highest quintile of triglycerides (≥ 160 mg/dl) had a 40%, 90%, and 4.8-fold increase in the risk of biliary stones, gallbladder cancer, and bile duct cancer, respectively, compared to the reference group (third quintile: 90–124 mg/dl) [9]. By contrast, Borena et al. found that there was no significant association between serum triglyceride level and gallbladder cancer [36]. The inconsistences might be ascribed to several reasons: (i) different study design and study participants; (ii) lipid measurement methods; (iii) lipid levels varied with times even in the same person; and (iv) inadequate adjustment for confounders. Given the inherent limitations of observational study, results from studies using genetic information might be an optimal complement for observational studies. For instance, Andreotti et al. reported that genetic variants in the lipid metabolism pathway (e.g., T allele of *LDLR* rs1003723) contribute to the risk of biliary tract stones and cancers, particularly of the bile duct [37]. Xu et al. reported that variants in a lipid metabolism-related gene (*ABCG8* rs11887534) was also associated with an increased risk of BtC [8]. However, these studies conventionally investigated effect of a single genetic variant on BtC risk alone. The additive effect of other variants was not taken into account.

In our study, we retrieved the GWAS summary statistics regarding blood lipid traits and BtC from East Asian populations owing to BtC were more commonly diagnosed in populations in East Asian countries and there was lack of large scale GWAS of BtC in other populations [2]. Herein, we tested associations between a total of four lipids and BtC risk leveraging MR analyses with a set of genetic variants as instruments. Paradoxical to the observational studies that reported high levels of serum triglycerides and low level of HDL were associated with risk of BtC [9, 38], we observed an inverse association between genetically determined level of triglyceride and BtC risk, whereas no significant association between HDL level and BtC risk was detected. Moreover, multivariable MR results suggested that genetically high LDL level was associated with an increased risk of BtC. This association, to our knowledge, was rarely reported in previous studies. In a cross-sectional study, the authors reported a putatively “U-shaped” association between LDL level and BtC risk [9].

Although some of our results are seemed to be contradictory with the generally accepted association, these findings were further validated in sensitivity analyses that with different assumptions. The genetic instruments that we used in the current study were free of weak instrumental variable bias and therefore could serve as strong indicators for circulating lipid levels. Furthermore, to ensure the robustness of results, we constructed a frame work of MR analysis to avoid the influences of heterogeneity and pleiotropy. The detected links between lipids and BtC risk are clues for future studies, although our study lacks ability to provide more explanations regarding the main findings.

The limitations of our study should be noted here. First, our results were based on genetic data from East Asian populations, which limited the possibility of extrapolation to other populations. Second, the exposure and outcome studies in two-sample MR analysis should not involve overlapping participants. The participants in BBJ and AGEN might to some extent overlapped. However, Japanese participants only accounted for approximate 7% of AGEN population. Third, the genetic data of BtC were derived from GWAS with limited number of cancer cases, which might introduce bias into GWAS results due to unbalanced case–control ratios. Larger GWAS will allow for more precision in the estimates of SNPs used as instruments in future MR. Fourth, our estimates might also subject to the inherent pitfalls of MR analysis such as selection bias [39]. Genetic variants which are related to specific phenotypes might also related to participation. For example, participants with high polygenic risk score for the circulating lipids might be more likely to drop-out in the cohort because they might be more susceptible to diseases such as chronic cardiovascular disease than those have low genetic risk of lipid traits. Moreover, the MR estimates might be confounded by other unobserved environmental factors [40]. For example, in our study, we cannot correct the effect of lipid-lowering medicine, and the circulating levels of lipids are susceptible to transitory fluctuations due to many reasons. These potential factors might bias the GWAS results of circulating lipids. Finally, all the results from IVW method were underpowered (< 80%), although we conducted a rigorous quality-control process. Further investigations with larger sample size on associations between circulating lipids and BtC risk are needed.

In conclusion, according to both univariate and multivariate MR estimates, genetically determined higher triglyceride level is associated with lower risk of BtC. On the contrary, genetically elevated LDL concentration is associated with higher risk of BtC according to multivariate MR estimate. Our findings suggest that circulating lipids may have roles in the development of BtC and have potentials to be prediagnostic biomarkers for BtC.

## Supplementary Information

**Additional file 1:** **Table S1.**The genetic instruments used in Mendelian analysis for high-density lipoproteincholesterol. **Table S2.** The geneticinstruments used in Mendelian analysis for low-density lipoprotein cholesterol.**Table S3.** The genetic instrumentsused in Mendelian analysis for total cholesterol. **Table S4.** The genetic instruments used in Mendelian analysis fortriglyceride. **Table S5.** Associationof biliary tract cancer with levels of circulating lipids according todifferent. **Figure S1.** The forestplot of leave-one-out analysis for high-density lipoprotein cholesterol. **Figure S2.** The forest plot ofleave-one-out analysis for low-density lipoprotein cholesterol. **Figure S3.** The forest plot of leave-one-outanalysis for total cholesterol. **FigureS4.** The forest plot of leave-one-out analysis for triglyceride. **Figure S5.** Results of pairwisemultivariable Mendelian randomization analysis. **Figure S6.** Results of multivariable Mendelian randomizationanalysis.

## Data Availability

The datasets generated and/or analysed during the current study are available in the following repository: GWAS summary data of circulating lipids were available on the Asian Genetic Epidemiology Network website (AGEN; https://blog.nus.edu.sg/agen/). GWAS summary data of biliary tract cancer were available on IEU Open GWAS project website (https://pheweb.jp/pheno/BtC).

## Abbreviations

BtC: Biliary tract cancer
HDL: High-density lipoprotein cholesterol
LDL: Low-density lipoprotein (HDL) cholesterol
CHL: Cholesterol
TRG: Triglyceride
MR: Mendelian Randomization
GWAS: Genome-wide association study
IVW: Inverse variance weighting
