# Supplementary material for: Mendelian randomization study of circulating lipids and biliary tract cancer among East Asians
Source: BMC Cancer. 2022 Mar 15;22:273. doi: 10.1186/s12885-022-09382-x (PMC8922750; doi:10.1186/s12885-022-09382-x)

**Supplementary materials**

**Mendelian randomization study of circulating lipids and biliary tract cancer among East Asians**

Table of content

[Table S1. The genetic instruments used in Mendelian analysis for high-density lipoprotein cholesterol. 2](#_Toc87599968)

[Table S2. The genetic instruments used in Mendelian analysis for low-density lipoprotein cholesterol. 4](#_Toc87599969)

[Table S3. The genetic instruments used in Mendelian analysis for total cholesterol. 5](#_Toc87599970)

[Table S4. The genetic instruments used in Mendelian analysis for triglyceride. 7](#_Toc87599971)

[Table S5. Association of biliary tract cancer with levels of circulating lipids according to different methods. 8](#_Toc87599972)

[Figure S1. The forest plot of leave-one-out analysis for high-density lipoprotein cholesterol. 9](#_Toc87599973)

[Figure S2. The forest plot of leave-one-out analysis for low-density lipoprotein cholesterol. 10](#_Toc87599974)

[Figure S3. The forest plot of leave-one-out analysis for total cholesterol. 11](#_Toc87599975)

[Figure S4. The forest plot of leave-one-out analysis for triglyceride. 12](#_Toc87599976)

[Figure S5. Results of pairwise multivariable Mendelian randomization analysis. 13](#_Toc87599977)

[Figure S6. Results of multivariable Mendelian randomization analysis. 13](#_Toc87599978)

Table S1. The genetic instruments used in Mendelian analysis for high-density lipoprotein cholesterol.

| SNP | CHR | position | Nearest genes# | effect_allele | other_allele | beta.exposure | se.exposure | beta.outcome | se.outcome |
| --- | --- | --- | --- | --- | --- | --- | --- | --- | --- |
| rs1011685 | 8 | 19830769 | *LPL* | T | C | 0.1664 | 0.0131 | 0.112749 | 0.115902 |
| rs1109166 | 16 | 67977382 | *PSKH1,CTRL,PSMB10,LCAT,SLC12A4* | T | C | -0.0749 | 0.0118 | -0.12981 | 0.107547 |
| rs11216126 | 11 | 116617240 | *BUD13* | A | C | -0.117 | 0.0091 | 0.025548 | 0.106533 |
| rs12708980 | 16 | 57012379 | *CETP* | T | G | 0.1536 | 0.0115 | 0.173321 | 0.150063 |
| rs1532085 | 15 | 58683366 |  | A | G | 0.1152 | 0.0092 | 0.07156 | 0.077981 |
| rs17138358 | 7 | 17920253 | *SNX13* | C | G | -0.042 | 0.0077 | -0.01327 | 0.077489 |
| rs17519093 | 11 | 116629905 | *BUD13,ZPR1* | A | G | 0.1152 | 0.015 | 0.123932 | 0.18698 |
| rs17821274 | 15 | 58684478 |  | T | C | -0.0547 | 0.0079 | 0.088185 | 0.079001 |
| rs1800588 | 15 | 58723675 | *LIPC,LOC101928694* | T | C | 0.137 | 0.0099 | -0.01673 | 0.076804 |
| rs1883023 | 9 | 107564846 | *ABCA1* | T | C | -0.061 | 0.008 | -0.07773 | 0.078256 |
| rs1883025 | 9 | 107664301 | *ABCA1* | T | C | -0.0983 | 0.0091 | 0.106465 | 0.085198 |
| rs1943973 | 18 | 47179516 |  | A | G | 0.082 | 0.0103 | -0.06237 | 0.107398 |
| rs1973688 | 15 | 58582540 |  | T | C | -0.087 | 0.0098 | -0.14503 | 0.097418 |
| rs2000813 | 18 | 47093864 | *LIPG* | T | C | 0.0543 | 0.0097 | 0.046731 | 0.088444 |
| rs2144300 | 1 | 230294916 | *GALNT2* | T | C | 0.0564 | 0.0104 | -0.06927 | 0.098234 |
| rs2297400 | 9 | 107599481 | *ABCA1* | T | C | -0.065 | 0.0088 | 0.036517 | 0.079708 |
| rs3760782 | 19 | 11346550 | *DOCK6,C19orf80* | T | C | -0.0676 | 0.0107 | -0.11545 | 0.089654 |
| rs3764261 | 16 | 56993324 | *HERPUD1,CETP* | A | C | 0.262 | 0.0141 | 0.078876 | 0.094707 |
| rs3786247 | 18 | 47118923 | *LIPG* | T | G | -0.0957 | 0.0102 | 0.110171 | 0.077015 |
| rs4420638 | 19 | 45422946 | *TOMM40,APOE,APOC1,APOC1P1* | A | G | 0.1129 | 0.0132 | 0.10342 | 0.143481 |
| rs445925 | 19 | 45415640 | *TOMM40,APOE,APOC1,APOC1P1* | A | G | 0.1197 | 0.0207 | -0.28181 | 0.154736 |
| rs4821116 | 22 | 21973319 | *UBE2L3,YDJC,CCDC116* | T | C | -0.0444 | 0.008 | 0.020328 | 0.080262 |
| rs6124760 | 20 | 44581453 | *PCIF1,ZNF335* | C | G | 0.1043 | 0.0168 | 0.019221 | 0.161578 |
| rs651821 | 11 | 116662579 | *BUD13,ZPR1,APOA5* | T | C | 0.1888 | 0.0102 | 0.200154 | 0.082798 |
| rs7115583 | 11 | 116784376 | *SIK3* | T | G | 0.0784 | 0.0115 | 0.105511 | 0.118701 |
| rs8034802 | 15 | 58724792 | *LIPC,LOC101928694* | A | T | 0.0824 | 0.0094 | -0.02033 | 0.081995 |

#: flanking distance (in kB) on each side was 20. NA denotes no gene was available.

Table S2. The genetic instruments used in Mendelian analysis for low-density lipoprotein cholesterol.

| SNP | CHR | Position | Nearest genes# | effect_allele | other_allele | beta.exposure | se.exposure | beta.outcome | se.outcome |
| --- | --- | --- | --- | --- | --- | --- | --- | --- | --- |
| rs10119 | 19 | 45406673 | *PVRL2,TOMM40,APOE,APOC1* | A | G | 0.2063 | 0.019 | -0.23501 | 0.127425 |
| rs10172650 | 2 | 21205457 | *APOB* | A | G | -0.0945 | 0.012 | 0.004845 | 0.120422 |
| rs12117661 | 1 | 55487346 | *BSND,PCSK9* | C | G | 0.0764 | 0.0141 | 0.115547 | 0.133346 |
| rs12610605 | 19 | 45370838 | *PVRL2* | A | G | 0.0495 | 0.0088 | 0.010751 | 0.076884 |
| rs12916 | 5 | 74656539 | *HMGCR,COL4A3BP* | T | C | -0.0793 | 0.0082 | -0.06848 | 0.076846 |
| rs12918956 | 16 | 72224335 | *PMFBP1* | T | C | 0.0503 | 0.0087 | -0.01473 | 0.077593 |
| rs174533 | 11 | 61549025 | *MYRF,TMEM258,MIR611,FEN1,FADS1* | A | G | -0.0602 | 0.0104 | -0.10152 | 0.0789 |
| rs1799955 | 13 | 32929232 | *BRCA2* | A | G | 0.0517 | 0.0087 | 0.017321 | 0.076968 |
| rs1865063 | 19 | 11341029 | *DOCK6,C19orf80* | T | C | -0.0549 | 0.0095 | -0.11334 | 0.08946 |
| rs2738452 | 19 | 11229218 | *LDLR,MIR6886* | A | G | -0.0734 | 0.0116 | -0.02992 | 0.115503 |
| rs2738464 | 19 | 11242307 | *LDLR,MIR6886,SPC24* | C | G | 0.0957 | 0.012 | 0.140272 | 0.082853 |
| rs2980869 | 8 | 126488250 |  | T | C | -0.0503 | 0.0084 | -0.06524 | 0.076824 |
| rs445925 | 19 | 45415640 | *TOMM40,APOE,APOC1,APOC1P1* | A | G | -0.5219 | 0.0214 | -0.28181 | 0.154736 |
| rs505151 | 1 | 55529187 | *PCSK9,USP24* | A | G | -0.104 | 0.0184 | 0.138774 | 0.182896 |
| rs5744651 | 5 | 74871997 | *POLK* | A | G | 0.1367 | 0.0228 | 0.230642 | 0.166838 |
| rs579459 | 9 | 136154168 | *ABO* | T | C | -0.0568 | 0.0096 | 0.144194 | 0.085974 |
| rs588245 | 2 | 21270057 | *APOB* | A | G | 0.074 | 0.0132 | 0.205052 | 0.137286 |
| rs599839 | 1 | 109822166 | *CELSR2,PSRC1,MYBPHL* | A | G | 0.1877 | 0.0162 | -0.03768 | 0.144532 |
| rs8051431 | 16 | 72015251 | *PKD1L3* | C | G | 0.0753 | 0.0093 | -0.06694 | 0.084031 |

#: flanking distance (in kB) on each side was 20. NA denotes no gene was available.

Table S3. The genetic instruments used in Mendelian analysis for total cholesterol.

| SNP | CHR | Position | Nearest genes# | effect_allele | other_allele | beta.exposure | se.exposure | beta.outcome | se.outcome |
| --- | --- | --- | --- | --- | --- | --- | --- | --- | --- |
| rs10119 | 19 | 45406673 | *PVRL2,TOMM40,APOE,APOC1* | A | G | 0.1357 | 0.0189 | -0.23501 | 0.127425 |
| rs10172650 | 2 | 21205457 | *APOB* | A | G | -0.0973 | 0.012 | 0.004845 | 0.120422 |
| rs12916 | 5 | 74656539 | *HMGCR,COL4A3BP* | T | C | -0.0794 | 0.0081 | -0.06848 | 0.076846 |
| rs12918956 | 16 | 72224335 | *PMFBP1* | T | C | 0.0513 | 0.0087 | -0.01473 | 0.077593 |
| rs1532085 | 15 | 58683366 |  | A | G | 0.0588 | 0.0099 | 0.07156 | 0.077981 |
| rs17122278 | 11 | 118449370 | *IFT46,ARCN1* | A | G | -0.0469 | 0.0088 | -0.00938 | 0.077496 |
| rs174533 | 11 | 61549025 | *MYRF,TMEM258,MIR611,FEN1,FADS1* | A | G | -0.0549 | 0.0103 | -0.10152 | 0.0789 |
| rs1800774 | 16 | 57015545 | *CETP* | T | C | -0.0688 | 0.0125 | -0.19464 | 0.156448 |
| rs1865063 | 19 | 11341029 | *DOCK6,C19orf80* | T | C | -0.0775 | 0.0095 | -0.11334 | 0.08946 |
| rs1883025 | 9 | 107664301 | *ABCA1* | T | C | -0.0735 | 0.0096 | 0.106465 | 0.085198 |
| rs2070895 | 15 | 58723939 | *LIPC,LOC101928694* | A | G | 0.0757 | 0.0108 | -0.01236 | 0.076917 |
| rs2297400 | 9 | 107599481 | *ABCA1* | T | C | -0.0577 | 0.0093 | 0.036517 | 0.079708 |
| rs2738452 | 19 | 11229218 | *LDLR,MIR6886* | A | G | -0.0688 | 0.0116 | -0.02992 | 0.115503 |
| rs2738464 | 19 | 11242307 | *LDLR,MIR6886,SPC24* | C | G | 0.0827 | 0.0119 | 0.140272 | 0.082853 |
| rs2980869 | 8 | 126488250 |  | T | C | -0.0705 | 0.0084 | -0.06524 | 0.076824 |
| rs3764261 | 16 | 56993324 | *HERPUD1,CETP* | A | C | 0.0809 | 0.0148 | 0.078876 | 0.094707 |
| rs579459 | 9 | 136154168 | *ABO* | T | C | -0.0588 | 0.0096 | 0.144194 | 0.085974 |
| rs599839 | 1 | 109822166 | *CELSR2,PSRC1,MYBPHL* | A | G | 0.1684 | 0.0161 | -0.03768 | 0.144532 |
| rs7192750 | 16 | 72014782 | *PKD1L3* | T | C | -0.0683 | 0.0092 | 0.067094 | 0.084043 |
| rs7254892 | 19 | 45389596 | *PVRL2,TOMM40,APOE* | A | G | -0.3981 | 0.0264 | -0.35756 | 0.201697 |
| rs7776054 | 6 | 135418916 |  | A | G | 0.0598 | 0.0093 | 0.131965 | 0.080583 |
| rs780092 | 2 | 27743154 | *GCKR* | A | G | 0.0526 | 0.0087 | 0.017496 | 0.084062 |
| rs9958734 | 18 | 47118398 | *LIPG* | T | C | -0.0919 | 0.0111 | 0.0929 | 0.077236 |

#: flanking distance (in kB) on each side was 20. NA denotes no gene was available.

Table S4. The genetic instruments used in Mendelian analysis for triglyceride.

| SNP | CHR | Position | Nearest genes# | effect_allele | other_allele | beta.exposure | se.exposure | beta.outcome | se.outcome |
| --- | --- | --- | --- | --- | --- | --- | --- | --- | --- |
| rs1077834 | 15 | 58723479 | *LIPC,LOC101928694* | T | C | -0.0843 | 0.0116 | 0.013713 | 0.077345 |
| rs11216206 | 11 | 1.17E+08 | *SIK3* | C | G | 0.0804 | 0.0105 | -0.18362 | 0.086535 |
| rs13233571 | 7 | 72971231 | *BCL7B,TBL2* | T | C | -0.1189 | 0.0141 | -0.0957 | 0.12851 |
| rs157582 | 19 | 45396219 | *PVRL2,TOMM40,APOE* | T | C | 0.1073 | 0.0168 | -0.13534 | 0.089198 |
| rs17482753 | 8 | 19832646 | *LPL* | T | G | -0.154 | 0.0144 | 0.113639 | 0.115885 |
| rs2001945 | 8 | 1.26E+08 |  | C | G | -0.0647 | 0.0087 | 0.019545 | 0.077868 |
| rs662799 | 11 | 1.17E+08 | *BUD13,ZPR1,APOA5* | A | G | -0.2825 | 0.0116 | 0.20139 | 0.082968 |
| rs7164909 | 15 | 58686754 |  | T | C | -0.0638 | 0.0111 | -0.00599 | 0.086438 |
| rs780094 | 2 | 27741237 | *GCKR* | T | C | 0.1047 | 0.0087 | -0.01964 | 0.077378 |
| rs995000 | 1 | 63107526 | *DOCK7* | T | C | -0.0801 | 0.0109 | 0.099126 | 0.110095 |

#: flanking distance (in kB) on each side was 20. NA denotes no gene was available.

Table S5. Association of biliary tract cancer with levels of circulating lipids according to different methods.

|  | HDL | LDL | CHL | TRG |
| --- | --- | --- | --- | --- |
| Inverse variance weighted | |  |  |  |
| *β* (se; *P* value) | -0.012 (0.007; 0.123) | -0.001 (0.008; 0.915) | -0.0001 (0.008; 0.989) | 0.001 (0.009; 0.936) |
| Q statistics (*P* value) | 2.77 (0.948) | 8.28 (0.407) | 8.19 (0.415) | 4.31 (0.828) |
| MR-egger |  |  |  |  |
| *β* (se; *P* value) | -0.027 (0.032; 0.423) | -0.001 (0.035; 0.987) | 0.024 (0.035; 0.512) | 0.007 (0.037; 0.849) |
| Q statistics (*P* value) | 3.02 (0.963) | 8.28 (0.506) | 8.70 (0.465) | 4.34 (0.887) |
| Intercept (*P* value) | 0.007 (0.636) | -0.0001 (0.993) | -0.011 (0.499) | -0.003 (0.860) |
| Weighted median |  |  |  |  |
| *β* (se; *P* value) | -0.012 (0.013; 0.414) | -0.003 (0.017; 0.856) | -0.007 (0.015; 0.670) | 0.010 (0.017; 0.585) |
| Weighted mode |  |  |  |  |
| *β* (se; *P* value) | -0.011 (0.010; 0.285) | -0.002 (0.011; 0.856) | -0.001 (0.011; 0.908) | 0.006 (0.011; 0.606) |

HDL, high density lipoprotein; LDL, low density lipoprotein; CHL, cholesterol; TRG, triglyceride.

Figure S1. The forest plot of leave-one-out analysis for high-density lipoprotein cholesterol.


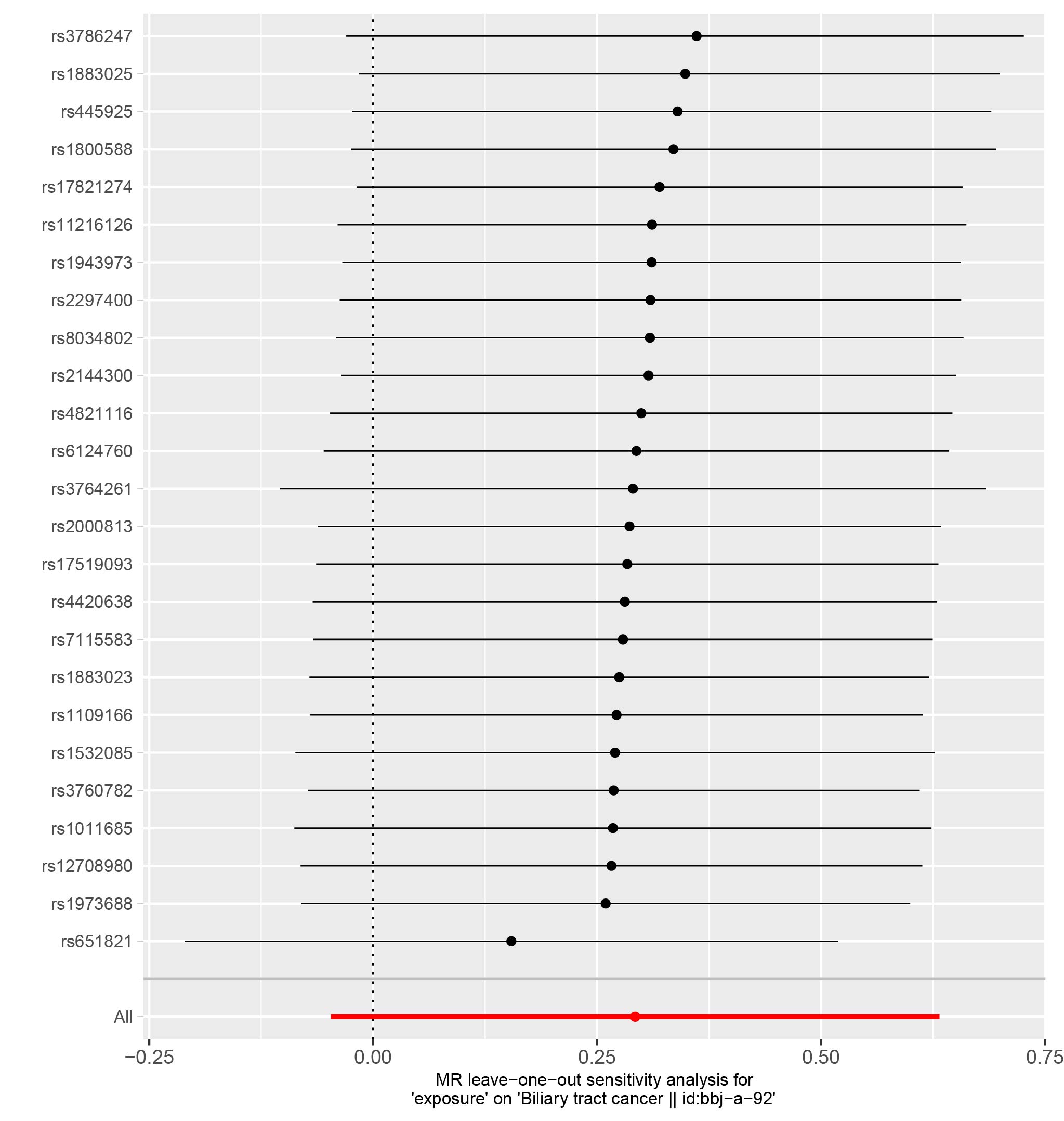


Figure S2. The forest plot of leave-one-out analysis for low-density lipoprotein cholesterol.


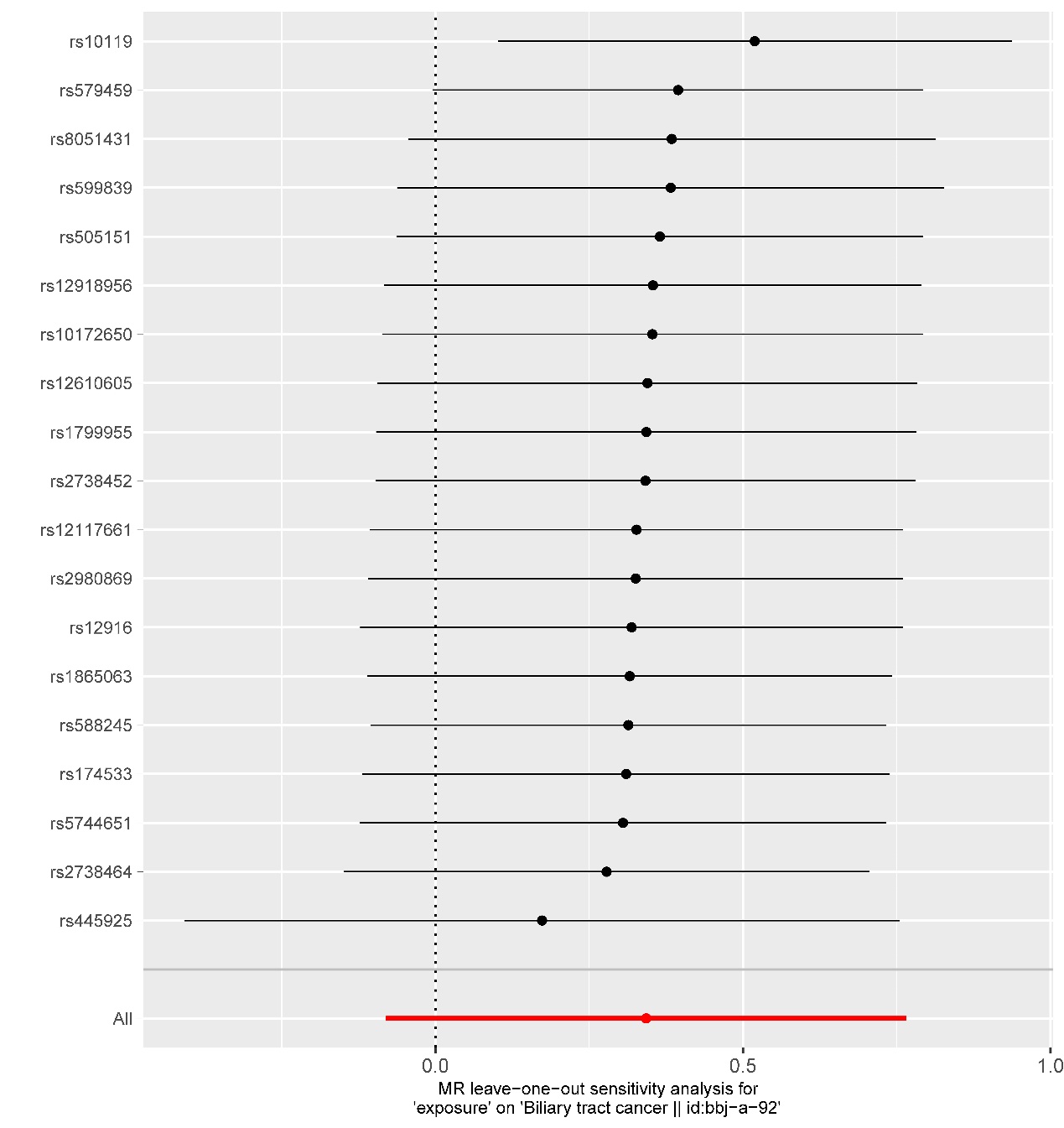


Figure S3. The forest plot of leave-one-out analysis for total cholesterol.


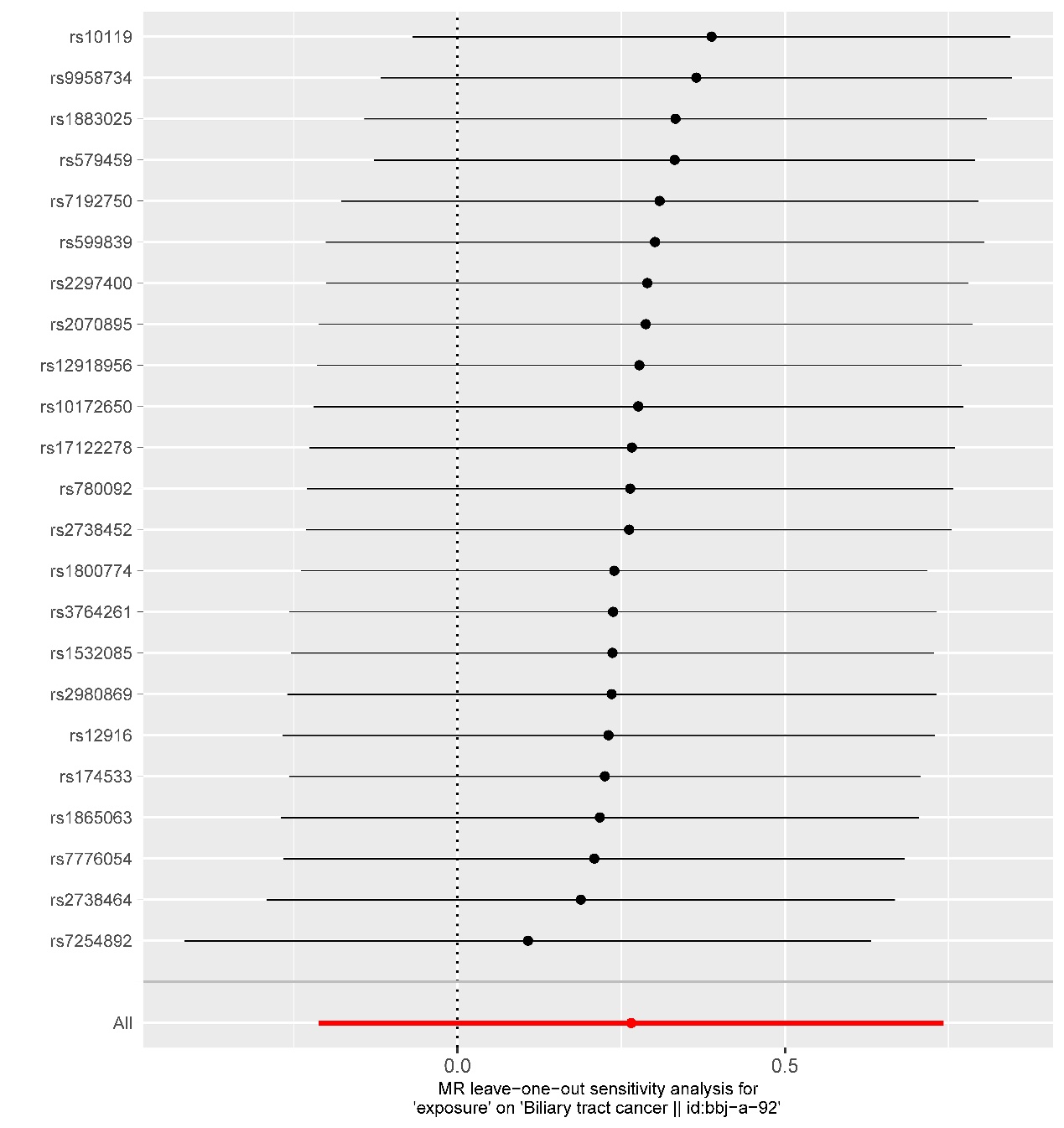


Figure S4. The forest plot of leave-one-out analysis for triglyceride.


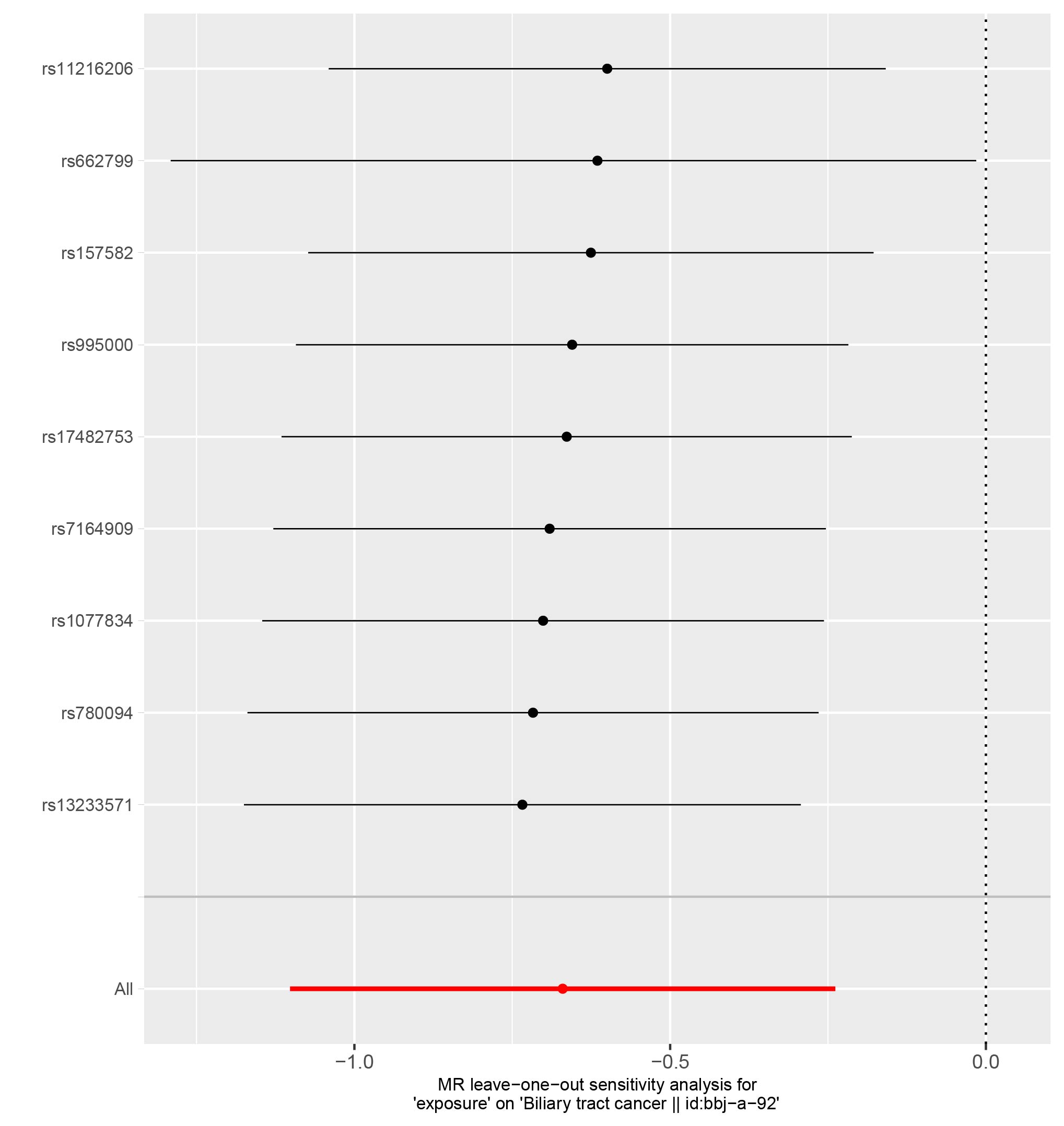


Figure S5. Results of pairwise multivariable Mendelian randomization analysis.


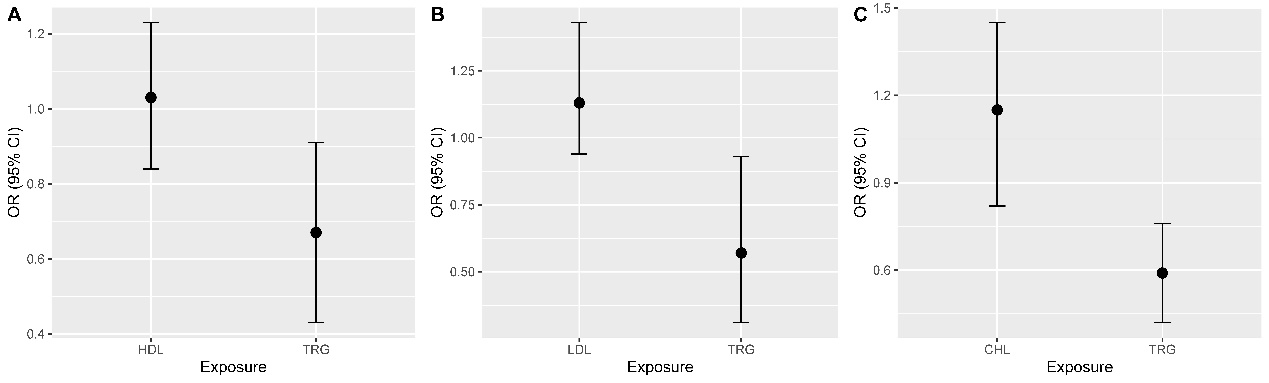


Figure S6. Results of multivariable Mendelian randomization analysis.


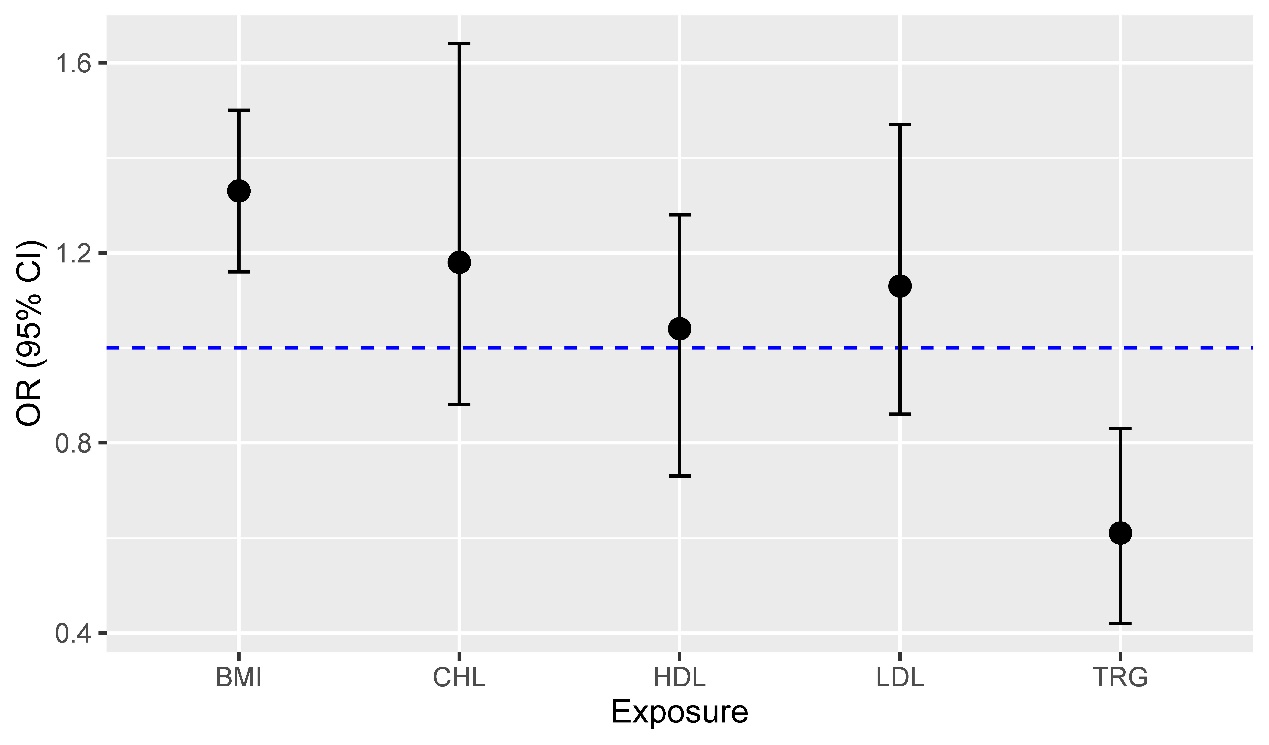

Supplement: Supplementary file 1 — Additional file 1: Table S1.The genetic instruments used in Mendelian analysis for high-density lipoproteincholesterol. Table S2. The geneticinstruments used in Mendelian analysis for low-density lipoprotein cholesterol.Table S3. The genetic instrumentsused in Mendelian analysis for total cholesterol. Table S4. The genetic instruments used in Mendelian analysis fortriglyceride. Table S5. Associationof biliary tract cancer with levels of circulating lipids according todifferent. Figure S1. The forestplot of leave-one-out analysis for high-density lipoprotein cholesterol. Figure S2. The forest plot ofleave-one-out analysis for low-density lipoprotein cholesterol. Figure S3. The forest plot of leave-one-outanalysis for total cholesterol. FigureS4. The forest plot of leave-one-out analysis for triglyceride. Figure S5. Results of pairwisemultivariable Mendelian randomization analysis. Figure S6. Results of multivariable Mendelian randomizationanalysis. [file 12885_2022_9382_MOESM1_ESM.docx]
